# Use of Paravertebral Block as an Alternative to General Anesthesia for Breast Surgeries: A Randomized Control Study

**DOI:** 10.7759/cureus.18322

**Published:** 2021-09-27

**Authors:** Navya Mishra, Ekramul Haque, Manisha Bhagat, Vishwanath Kumar, Usha Suwalka, Piu Gorai

**Affiliations:** 1 Anaesthesiology, Rajendra Institute of Medical Sciences, Ranchi, IND

**Keywords:** post operative pain, unilateral breast surgery, analgesia cancer-related breast surgery, pain on vas, paravertebral block (pvb)

## Abstract

Background: General anaesthesia (GA) is the conventional technique used for surgical treatment of breast lumps. However, various side effects and complications of GA, such as postoperative pain, nausea, vomiting, and increased hospital stay increase morbidity in patients. Regional anaesthesia using multiple injection paravertebral block is an ideal alternative to GA for breast surgeries.

Methods: Sixty female patients posted for unilateral breast surgery were randomly divided into two groups, Group P for paravertebral block and group G for GA, and compared on the basis of time taken for induction of anaesthesia, postoperative pain relief on basis of Visual Analogue Scale (VAS) score, postoperative nausea and vomiting (PONV) and duration of hospital stay.

Results: Duration of surgery in group P was 64.75±18.07 and 67.32±17.64 in group G respectively (P>0.05). Time for inducing anaesthesia was significantly longer in group P (17.15±3.92min) compared to group G (5.90±1.75min) with P<0.05. Significant difference (P<0.001) was observed in the mean duration of postoperative analgesia of group P (298.34±67.02min) and group G (107.68±27.28min). The VAS scores in immediate postoperative period and after two and four hours in the postoperative period were significantly higher in group G (P<0.05). The incidence of postoperative nausea and vomiting was significantly higher in group G (13 out of 30 patients) than group P (four out of 28 patients) with P<0.05.

Conclusion: The efficacy and safety of paravertebral block for operative treatment of breast tumors, excellent analgesia in early postoperative period, requirement of significantly lesser amount of postoperative analgesics, decreased incidence of PONV and negligible complications along with early ambulation and hospital discharge makes it an afferent cost-effective block of choice for unilateral breast surgeries.

## Introduction

Breast surgeries are very common in India, which has a high incidence rate of breast cancer (25.8 per 1,00,000 women) and fibroadenoma breast (45.5%) [1-2]. General anesthesia (GA) has always been the conventional technique used for surgery of breast lumps. However, various side effects and complications of GA were noted such as postoperative pain, nausea, and vomiting in patients. Regional anaesthesia using multiple injection paravertebral block (PVB) is an ideal alternative to GA for breast surgeries. Its benefits include prolonged postoperative pain relief, reduction in postoperative nausea and vomiting and early ambulation of the patient. The aim of our study was to compare the efficacy of thoracic PVB as an anesthetic procedure for elective breast lump surgeries with GA, duration of postoperative pain relief being the primary objective.

## Materials and methods

After the approval obtained by the Institutional Review Board of Rajendra Institute of Medical Sciences (RIMS) ethical committee (104,10/4), written consent was obtained from 60 female patients aged 18-65 years, ASA physical status I and II scheduled for unilateral breast surgery without axillary extension who were included in our randomized clinical study. Patients with ASA grade III and IV, age <18 or >65 years, and those who refused to give consent were excluded from the study. Estimating an increase in postoperative analgesia duration of 30% to be relevant clinically, by calculating a power of 80% (beta=0.2) at 0.05 level of significance (alpha=0.05) a minimum of 25 patients in each group were required for our study. To be on the safe side considering patient dropouts and procedure failure, 30 patients in each group were taken in our study. On the day of surgery patients were randomized by using sealed envelopes into group P (n=30) and group G (n=30) to receive either thoracic paravertebral block or GA. All monitors were attached to the patient in the operation theater and an intravenous line was secured, ringer lactate was given intravenous and preoperative vitals (blood pressure, heart rate, and blood oxygen saturation [SpO2]) were recorded.

**Figure 1:**
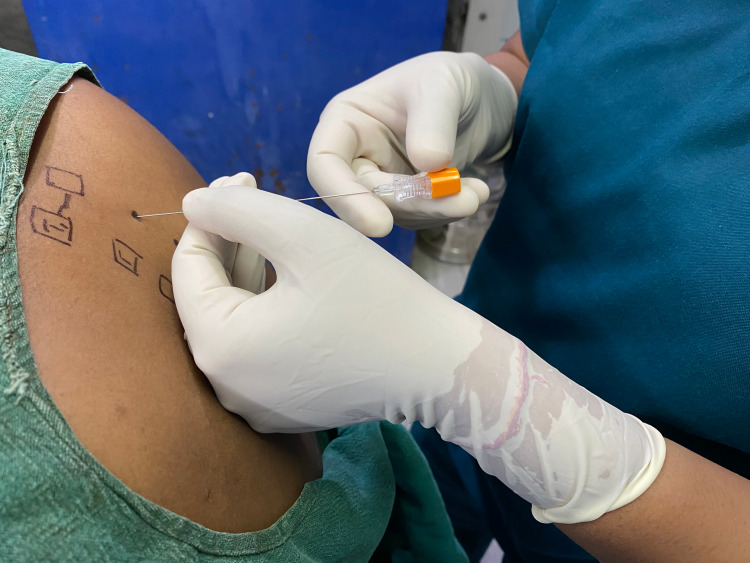
25 G needle inserted at T3 level.

After explaining the anesthetic procedure to the patients, group P patients were seated for placement of block. Thoracic paravertebral block was then performed according to the technique described by Moore and Katz [3-4]. The superior aspect of the spinous process of C7-T6 were marked as shown in Figure 1 and skin entry points were 2.5 cm lateral to the marks. Lidocaine (2%) was administered intradermally at the site of needle insertion for local anaesthesia. A 25-G Quincke spinal needle was inserted perpendicular to the skin and advanced 2 to 4 cm in order to approach the transverse process. After coming in contact with the transverse process the needle was then withdrawn directed caudal to the transverse process advanced further 1.5 to 2 cm till loss of resistance was experienced as explained by Eason and Wyatt [5]. Loss of resistance is experienced when the needle pierces the superior costotransverse ligament to enter into the thoracic paravertebral space [6]. After aspiration, 3 to 4 ml of local anaesthetic mixture (bupivacaine 0.5% and lidocaine 2%) was administered per level by attaching a syringe through extension tubing containing local anaesthetic (5 ml lidocaine 2% and 15 ml bupivacaine 0.5%). Time for performance of block and onset of sensory loss was noted. A block was considered inadequate if onset of pinprick discrimination was not evident within 15 minutes or failure to achieve adequate sensory block (T2-T6) within 20 minutes. In cases of failure patients were given GA, otherwise patients were then given intravenous injection glycopyrrolate 0.2 mg, injection midazolam 1 mg and injection fentanyl 25-50 mcg/kg for sedation.

The control group (G) patients were given preoperative intravenous injection glycopyrrolate 0.2 mg, injection midazolam 1 mg for sedation and injection fentanyl 2 mcg/kg was administered for analgesia, induction was done using injection propofol (2 mg/kg) and injection vecuronium (0.1 mg/kg), followed by intubation, for maintenance O2:N2O was given (3:2) isoflurane (0.8%) and vecuronium (0.06 mg/kg i.v.). Time for performance (starting from preoxygenation till completion of intubation) was noted. On completion of surgery intravenous injection neostigmine (40-70 mcg/kg) and glycopyrrolate (7-15 mcg/kg) were administered to all patients for reversal of neuromuscular blockade.

Duration of surgery was defined as the time interval from surgical incision to completion of dressing after closure of the wound. All patients were postoperative monitored in the recovery room for the first 24 hours. Assessment of pain was done just after shifting the patient to the recovery room and thereafter at two, four, six, 12 and 24 hours. Postoperative pain was assessed using standard Visual Analogue Scale (VAS) score of 0 to 10 (0=no pain, 10=unbearable pain). VAS scores >4 were administered rescue analgesia (injection tramadol 50 mg intravenous bolus repeated after 15 minutes if required). To prevent drug-induced nausea caused by tramadol, antiemetic (intravenous ondansetron 4 mg) was administered to every patient before administering injection tramadol. The total doses of tramadol given during the first 24-hour period was recorded. Duration of postoperative analgesia was defined as the time between the last suture application and first complaint of pain by the patient (VAS score >4). Assessment of postoperative nausea and vomiting (PONV) was done after completion of surgery and before administering first dose of antiemetic (injection ondansetron 4 mg i.v.) along with rescue analgesia (injection tramadol 50 mg i.v.) to avoid any controversy in our study due to drug-induced postoperative nausea vomiting caused by injection tramadol. The number of patients complaining of PONV were accounted and treated accordingly. Patients at the time of discharge were requested to give their feedback regarding overall experience of surgery following a numeric rating scale (NRS 0-100). Statistical analysis was performed using Fisher’s exact test, Chi-square test and Mann Whitney U test.

## Results

The study was conducted over a six-month time period. In two patients of group P we had to administer GA due to inadequate effect of block and they were excluded from the study whereas in group G all patients were successfully administered GA. Hence, data collection of only 58 patients were used for study analysis; group P (n=28) and group G (n=30). Procedure failure was already considered as a possibility while selecting sample size and appropriate sample size was selected to avoid conflicts in data study due to patient dropouts. No significant difference was found in the demographic pattern and preoperative vital parameters as shown in Table 1. In 28 patients of group P the PVB was itself sufficient for surgery and no added analgesia was required during the surgery. Duration of surgery in group P was 64.75±18.07 min and 67.32±17.64 min in group G respectively (P>0.05) as shown in Table 2. Time taken for induction of anaesthesia was significantly longer in group P (17.15±3.92 min) compared to group G (5.90±1.75 min) with P<0.05 (Table 2). No significant difference was observed in intraoperative vital parameters among the two groups (P>0.05). The duration of postoperative analgesia ranged from 120 to 416 min in group P and from 74 to 165 min in group G, therefore significant difference (P<0.001) was observed in the mean duration of postoperative analgesia of group P (298.34±67.02 min) and group G (107.68±27.28 min) as shown in Table 3. Immediate postoperative VAS score as well as at two and four hours postoperative period were significantly higher in group G (P<0.05) whereas VAS scores at six, 12 and 24 hours were similar in both groups as shown in Table 3. Total dose of rescue analgesic (injection tramadol) given during the first 24 hours was significantly higher in group G (194.62±32.46 mg) than group P (102.48±24.24 mg) with P<0.05. Complaints of postoperative nausea and vomiting after surgery and before administering antiemetic along with injection tramadol (rescue analgesia) were significantly higher in group G (13 out of 30 patients) than group P (four out of 28 patients) with P<0.05. The patient satisfaction score on the numerical scale of 0-100 at the time of discharge was 82.32±8.79 in group P and 73.61±8.23 in group G therefore significantly higher in group P patients than in group G patients (P<0.05).

**Table 1:** Demographic & pre-operative parameters MAP: Mean Arterial Pressure

Parameters	Group p ( n=28)	Group g (n=30)	P value
Age (years)	37.28±14.63	35.32 ± 15.09	0.92
Weight (Kg)	50.68 ± 7.62	49.82 ± 4.68	0.60
Height (cm)	152.88 ± 5.90	153.46 ±5.60	0.38
ASA status (I/II)^$^	18/10	20/10	0.76
Preoperative heart rate (bpm)	82.34 ± 6.88	79.32 ± 6.35	0.34
Preoperative MAP (mmHg)	90.14 ± 5.78	92.1 ± 8.32	0.14

**Table 2:** Intraoperative characteristics MAP: Mean Arterial Pressure

Parameters	Group p (n=28)	Group g (n=30)	P value
Induction time (mins)	17.15 ± 3.92	5.90 ± 1.75	Less than 0.05
Duration of surgery (mins)	64.75 ± 18.07	67.32 ± 17.64	0.76
Intraoperative pulse (bpm)	78.39 ± 6.25	79.20 ± 6.35	0.86
Intraoperative MAP (mmHg)	91.20 ± 6.88	90.34 ± 6.35	0.34

**Table 3:** Postoperative analgesia & PONV VAS: Visual Analogue Scale, PONV: Postoperative Nausea and Vomiting

Parameters	Group p (n=28)	Group g (n=30)	P value
Time to first analgesic at VAS >-4 (mins)	298.34 ± 67.02	107.68 ± 27.28	Less than 0.01
Total dose of rescue analgesic (tramadol)mg	102.48 ±24.24	194.62 ±32.46	Less than 0.05
VAS score in immediate post-operative period	0.12 ± 0.04	1.43 ± 0.76	Less than 0.01
VAS score at 2 hrs	0.38 ± 0.072	3.62 ±1.09	Less than 0.01
VAS score at 4 hrs	1.68 ± 0.71	3.84 ± 0.76	Less than 0.05
VAS score at 6 hrs	2.74 ± 0.78	2.86 ±0.47	0.13
VAS score at 12 hrs	3.12 ± 0.36	3.87 ± 0.34	0.58
VAS score at 24 hrs	3.47 ± 0.38	3.92 ±0.56	0.28
VAS score at first rescue analgesic	4.84 ± 0.46	4.98 ± 0.72	0.58
PONV requiring treatment; n (%)	4/28	13/30	Less than 0.05
Patient satisfaction score(0-100)	82.32±8.79	73.61±8.23	less than 0.05

Complications such as epidural spread, intravascular injection, hemodynamic instability, pneumothorax or persistent pain were not observed administering the block. No case of bilateral spread of block was recorded. Eleven patients complained of transient burning sensation over breast area after application of block.

## Discussion

Paravertebral block can be performed successfully and with minimal complication in patients undergoing selective breast surgeries [7-8]. It is ideal for day care surgeries such as fibroadenoma breast, breast lump removal as it provides adequate analgesia in the postoperative period as well and helps in early ambulation of the patient [7-9]. However, similar to a previous study [7], in our study the time of induction in group P was significantly higher than in group G. Similar to previous studies [9,10], in our study patients undergoing surgery using PVB technique showed lower incidence of postoperative nausea and vomiting whereas incidence of PONV in patients undergoing breast surgeries under GA was significantly high. Multiple PVB’s (from T1 to T6) level provide adequate anaesthesia and effective surgical manipulation for breast surgeries and in spite of multiple punctures, the patients were satisfied with this procedure as it provided adequate and prolonged pain relief both during and after surgery [11-12]. Postoperative pain control was assessed by the time to first analgesic consumption, total amount of analgesia consumed in the first 24 hours after surgery and VAS scores recorded after surgery at regular time intervals. Like the previous study [7], in our study also the time to first analgesic in the postoperative period was significantly greater in group P than in group G as PVB provides longer duration of postoperative analgesia. VAS scores just after surgery and up till four hours postoperative were significantly lower in group P than in group G. Previous study [7] also reported similar observations. Due to unavailability of patient-controlled analgesia (PCA) devices, we could not achieve significant reduced pain scores after six hours of surgery unlike the study by Klein and colleagues, who found reduced pain scores even 72 hours postoperative day [13]. In our study mean requirement of rescue analgesic in the first 24 hours was also much less in group P as compared to group G, similar results were observed in previous studies as well [7-9]. Similar to previous study [7], in our study also patients of group P were more satisfied and gave good reviews regarding overall intraoperative anaesthesia experience and postoperative pain relief at the time of discharge. In our study adequate effect of PVB was achieved in 28 out of 30 patients of group P whereas two patients were converted to GA due to failed block effect but in group G all patients were successfully administered GA. In previous study by Sabyasachi and colleagues [7] PVB was effective in 29 out of 30 patients and only one patient was converted to GA. Due to unavailability of an ultrasound machine we could not use ultrasound guidance for block administration, but the probability of inconsistent block can be easily reduced by using ultrasound-guided PVB technique as done in advanced centers.

Breast surgeries conducted under regional anaesthesia leads to early ambulation therefore early discharge of patient hence can reduce hospital stay cost as recently a study demonstrated an approximately 75% cost reduction in patients undergoing ambulatory breast surgery as opposed to surgery followed by two to three days hospitalization [14].

## Conclusions

To conclude, the efficacy and safety of paravertebral block for operative treatment of unilateral breast tumors, excellent analgesia in early postoperative period, requirement of significantly lesser amount of postoperative analgesics, decreased incidence of PONV and negligible complications along with early ambulation and hospital discharge makes it an afferent block of choice for unilateral breast surgeries and is considered the gold standard, although increased induction time and chances of block failure are two drawbacks of this technique, although the latter can be overcome to some extent by using advance techniques such as ultrasound guidance. 
